# Acoustical Enrichment during Early Development Improves Response Reliability in the Adult Auditory Cortex of the Rat

**DOI:** 10.1155/2018/5903720

**Published:** 2018-05-30

**Authors:** Zbyněk Bureš, Kateryna Pysanenko, Jiří Lindovský, Josef Syka

**Affiliations:** ^1^Department of Auditory Neuroscience, Institute of Experimental Medicine, Czech Academy of Sciences, Vídeňská 1083, 14220 Prague 4, Czech Republic; ^2^Department of Technical Studies, College of Polytechnics, Tolstého 16, 58601 Jihlava, Czech Republic

## Abstract

It is well known that auditory experience during early development shapes response properties of auditory cortex (AC) neurons, influencing, for example, tonotopical arrangement, response thresholds and strength, or frequency selectivity. Here, we show that rearing rat pups in a complex acoustically enriched environment leads to an increased reliability of responses of AC neurons, affecting both the rate and the temporal codes. For a repetitive stimulus, the neurons exhibit a lower spike count variance, indicating a more stable rate coding. At the level of individual spikes, the discharge patterns of individual neurons show a higher degree of similarity across stimulus repetitions. Furthermore, the neurons follow more precisely the temporal course of the stimulus, as manifested by improved phase-locking to temporally modulated sounds. The changes are persistent and present up to adulthood. The results document that besides basic alterations of receptive fields presented in our previous study, the acoustic environment during the critical period of postnatal development also leads to a decreased stochasticity and a higher reproducibility of neuronal spiking patterns.

## 1. Introduction

For the auditory system of rats, the early postnatal developmental period (the so-called critical period, CP) represents the epoch of increased plasticity and susceptibility [1–3]. Progressive refinements taking place during this time substantially modulate the structure and function of the auditory centers, often leaving a lifelong trace. An important message brought by recent research is that the final maturation of the system and development of its proper response characteristics are heavily influenced by ongoing auditory stimulation [4–6]. Interventions such as sensory deprivation, monotonous stimulation, or excessive sound exposure result in impairments of neuronal responsiveness both in the auditory cortex (AC) and in subcortical structures such as the inferior colliculus (IC) [5, 7–10]. On the other hand, a richer and stimulating environment may improve the response properties of the system by, for example, more favorable shaping of the neuronal receptive fields [6, 11, 12]. In the simplest case, when the augmented stimulation comprises an elementary sound like pure tone or click train, the outcome is usually characterized by selective enhancement of responses related to that specific stimulus [3, 4, 13]. A more beneficial situation, however, is when the responses of the auditory system are amended globally in a nonspecific manner. To achieve this, the enriched environment has to be more complex and possibly contain a positive feedback to motivate the animals to attend to the stimulation [6, 14, 15]. Our previous results have shown that application of a complex acoustically enriched environment (AEE) during the period of development nonselectively and permanently improves the representations of tonal frequency and intensity in IC and AC neurons by increasing their sensitivity and frequency selectivity and by lowering the number of nonmonotonic rate-intensity functions [6, 16]. Advancing one step further from the basic evaluation of neuronal receptive fields performed in our previous work, the aim of the current study is to address the ability of neurons to precisely encode a given stimulus using either the rate code or the temporal code. Employing mostly complex temporally modulated stimuli, we focus on the assessment of the reliability, stability, and temporal acuity of individual responses. By providing more stable and repeatable response patterns that could be more easily detected in a background spiking noise, the acoustical enrichment during the developmental period, complemented with an active stimulus-reward paradigm, may ultimately enhance the stimulus discrimination ability of the animals.

## 2. Materials and Methods

### 2.1. Experimental Groups

In total, 51 female rats were used in the experiment (strain Long-Evans, obtained from the local breeding facility). One group of rats (*n* = 27) denoted as *enriched* was exposed to an acoustically enriched environment reinforced with active feedback for two weeks starting on postnatal day 14. The acoustic environment was based on a broadband (983 Hz–48461 Hz) amplitude-modulated (low-pass exponential noise envelope with cut-off frequency 2 Hz) rippled noise (depth of the spectral ripples 30 dB) presented at 55 dB SPL. To draw the animals' attention to the overall acoustic stimulation, the continuous background was complemented with several types of randomly appearing embedded target sounds (60 dB SPL, 500 ms duration, spectral contents centered near 6 kHz): pure tone, sawtooth signal, sinusoidally frequency-modulated tone (modulation frequency 10 Hz), and 1/3-octave noise. The frequency-modulated tone triggered the release of reward, which was slightly delayed relative to the triggering sound. The drop was always available for approximately 2 s and then fell out of reach of the animals. The presence and identity of animals at the reward-delivering spout were monitored by a custom-made system based on an infrared gate (Coulbourn Instruments), an Arduino RFID reader, and a MATLAB script. The RFID chips (kindly donated by LUX-IDent, Czech Republic) were subcutaneously implanted on the neck under temporary isoflurane anesthesia. More details about the AEE can be found in [6, 16].

**Figure 1:**
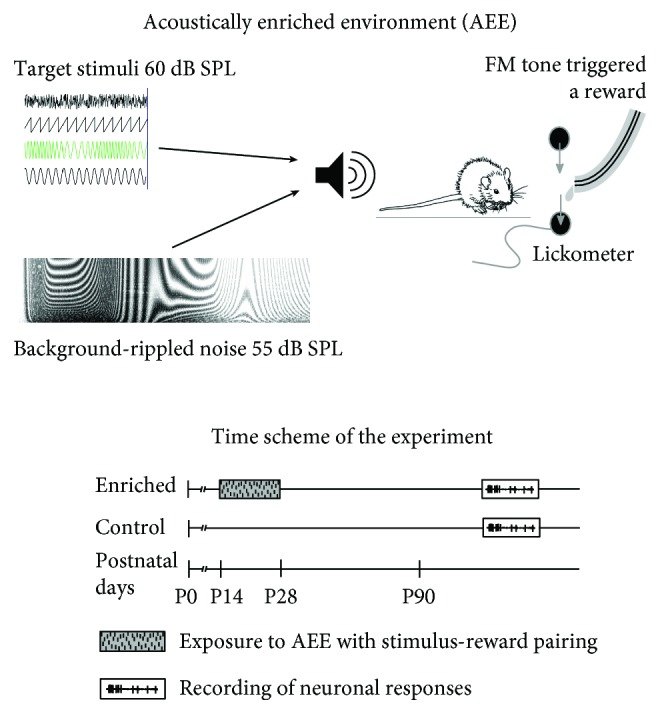
Schematic illustration of the experimental paradigm.

An age-matched group of rats denoted as controls (*n* = 24) was raised in standard conditions with no acoustical enrichment. The responses of AC neurons were recorded later, at the age of 3 to 6 months. A schematic illustration of the experimental paradigm is shown in Figure 1. The care and use of animals were approved by the Ethics Committee of the Institute of Experimental Medicine, Academy of Sciences of the Czech Republic, and followed the guidelines of the EU Directive 2010/63/EU for animal experiments.

### 2.2. Recording of the Neuronal Activity in the AC

Recording of the neuronal activity in the AC was carried out in a sound-proof anechoic room. The rats were anaesthetized with an intramuscular injection of a mixture of 35 mg/kg ketamine (Calypsol 50 mg/ml; Gedeon Richter, Budapest, Hungary) and 6 mg/kg xylazine (Xylapan 20 mg/ml; Vetoquinol SA., Lure Cedex, France). Supplementary subcutaneous injections of one-half of the original dose of the ketamine-xylazine mixture were administered approximately every hour to maintain a sufficient level of anesthesia. The body temperature was maintained at 37-38 deg C using a heating pad. Respiratory and heart rates, along with pedal reflex (toe pinch) or eye blink reflex, were monitored. The skin and muscles above the right temporal region of the skull were removed, and a craniotomy with a diameter of approximately 5 mm was performed above the AC area. The base of an S-shaped holder was screwed into the skull above the frontal part of the right hemisphere. After placing the rat in a stereotaxic apparatus and fixing the head with the previously screwed holder, the dura mater was removed. The A1 location was determined by recording tone-evoked responses at the cortical surface using a custom-made multichannel low-impedance electrode array. The responses of single neurons or small groups of adjacent neurons were recorded using a 16-channel electrode array (NeuroNexus Technologies, single shank probe, 50 *μ*m or 100 *μ*m distance between electrode spots) inserted into the AC using an electronic driver. The recording depths varied between 100 and 1200 *μ*m beneath the cortex surface, and the recordings thus originated from all layers of the AC. The amplified and bandpass-filtered (300 Hz–10 kHz) signal processed by a TDT System III, RX5-2 Pentusa Base Station was subsequently recorded and analyzed with BrainWare software (Tucker Davis Technologies, Alachua, FL, USA). The discrimination of individual action potentials was performed online based on amplitude thresholding.

### 2.3. Test Stimuli and Data Analyses

Acoustic stimuli were generated with a TDT System III using the RP 2.1 Enhanced Real-Time Processor and delivered in free-field conditions via a two-way loudspeaker system (Selenium 6W4P woofer and RAAL70-20 tweeter) placed 70 cm in front of the animal's head.

To quantify the stability and precision of the rate coding, we measured the variability of noise-evoked responses. White noise bursts (60 ms in duration, 5 ms rise/fall times) with a variable intensity (10 dB step) were presented in a random order, each stimulus appearing at least fifty times. For each stimulus, the variance of evoked spike rates was computed for each neuron. To better judge the level discrimination ability, detection distance at a given sound level was computed as follows:

(1) $$\begin{array}{c} d\left(I\right)=\frac{\mu \left(I+10\right)-\mu \left(I-10\right)}{\sigma \left(I\right)}, \end{array}$$

where *I* is the sound level in dB, *μ*(*I*) is the mean spike count at sound level *I*, and *σ*(*I*) is the standard deviation of the spike count at sound level *I*.

To assess the properties of the temporal coding, amplitude-modulated (AM) white noise, frequency-modulated (FM) tones, and a series of clicks were used. In the case of amplitude modulation, white noise bursts (500 ms in duration) were modulated by a sinusoidal envelope ranging from 0% to 100%. The modulation frequencies ranged from 2 Hz to 24 Hz with a 2 Hz step. The equivalent (time-averaged) SPL was set to 60 dB. In the case of frequency modulation, pure tone pulses (1000 ms in duration, frequency matching the best frequency of the measured neuron) were modulated sinusoidally with logarithmically spaced modulation frequencies ranging from 2 Hz to 100 Hz. The mean frequency shift was set to 0.23 octaves, and the equivalent SPL was set to 60 dB. In the case of click trains, short rectangular pulses (0.1 ms pulse duration, 2000 ms click train duration) were presented with a variable repetition rate. The repetition rate varied logarithmically from 2 Hz to 100 Hz. All three temporally structured stimuli (i.e., the AM noise, the FM tones, and the click trains) were used to determine the amount of synchronization of neuronal responses with the stimulus. Vector strength values along with the Rayleigh statistics were computed for each sound-evoked spike pattern; only responses having Rayleigh statistics of at least 5.991 were considered as significantly phase-locking [17]. The vector strength value quantifies how well a spike train is synchronized with a periodic stimulus waveform and is computed as follows:

(2) $$\begin{array}{c} VS=\frac{\sqrt{{\left(\sum_{i=1}^{n}\sin\theta_{i}\right)}^{2}+{\left(\sum_{i=1}^{n}\cos\theta_{i}\right)}^{2}}}{n}, \end{array}$$

where *n* is the number of spikes and θ_*i*_ is the phase of each spike, given as

(3) $$\begin{array}{c} \theta_{i}=2\pi \frac{t_{i}\mathrm{mod}T}{T}, \end{array}$$

where *t*_*i*_ is the spike time relative to the stimulus onset and *T* is the period of the modulation frequency (both in milliseconds). The Rayleigh statistics was computed as

(4) $$\begin{array}{c} R=2n{\left(VS\right)}^{2}. \end{array}$$

In addition, the FM tones and click trains were used to determine the similarity of spike trains evoked by the same stimulus. The van Rossum distance [18, 19] was computed for each pair of responses of the same neuron to the same stimulus. The van Rossum distance quantifies the similarity of two spike trains *X*(*k*) and *Y*(*k*) and is computed as follows: first, each discrete spike train is transformed to continuous function *x*(*t*) and *y*(*t*), respectively, using convolution of the spike train with exponential function.

(5) $$\begin{array}{c} H\left(t\right)\exp\left(-\frac{t}{\tau }\right), \end{array}$$

where *τ* is the time constant (set here to 10 ms) and *H*(*t*) is the Heaviside step function which is zero for *t* < 0 and one otherwise. Then, the van Rossum distance is computed as

(6) $$\begin{array}{c} D^{2}=\frac{1}{\tau }\int_{0}^{\infty }{\left[x\left(t\right)-y\left(t\right)\right]}^{2}dt. \end{array}$$

Both vector strength values and van Rossum distances were computed from the complete response (including onset and sustained portions of the evoked response).

### 2.4. Statistical Analysis

Normality of distributions of the data sets was tested using Shapiro-Wilk test prior to subsequent analyses. Due to the fact that many of the data sets were found to have distribution significantly deviating from normality, two-sided Wilcoxon rank sum tests were computed for comparisons of the medians of two data sets. Chi-square tests were employed to compare the percentual occurrences of certain phenomenon (e.g., percentages of phase-locking neurons). Possible relationship between synchronization index (vector strength) and response magnitude was tested using Spearman's linear correlation coefficient. In all cases, the alpha level was set to 0.05. Statistical analyses were performed using Prism (GraphPad Software Inc., La Jolla, California, USA) and MATLAB (Mathworks Inc., Massachusetts, USA).

## 3. Results

**Figure 2:**
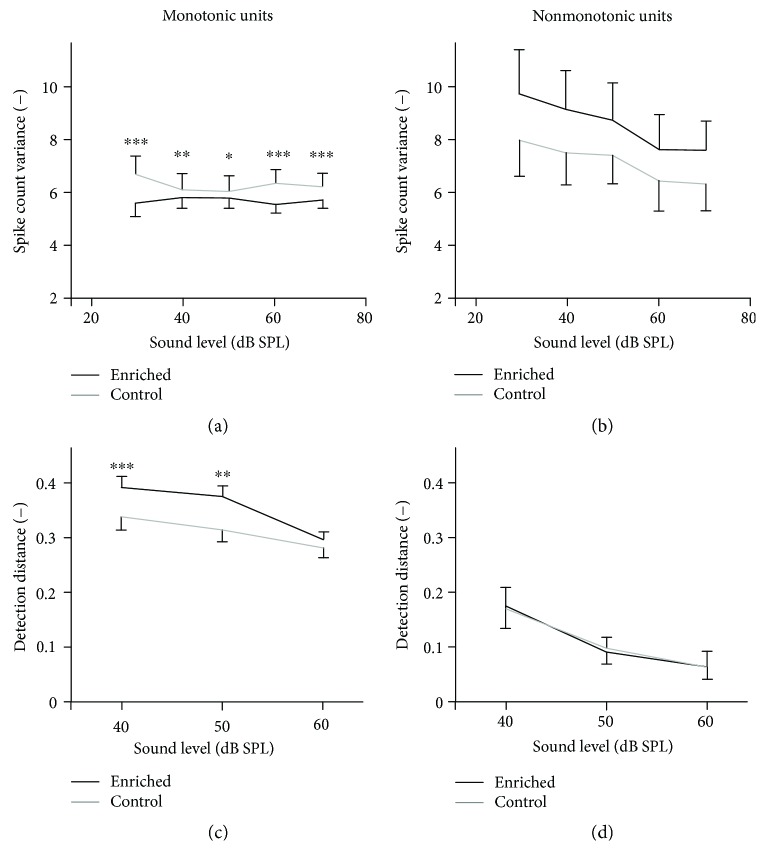
Spike count variances computed for noise-evoked responses in monotonic (a) and nonmonotonic (b) units. Detection distances computed for noise-evoked responses in monotonic (c) and nonmonotonic (d) units. The curves represent medians ±95% confidence intervals, and significant differences of individual data pairs are marked with asterisks (Wilcoxon rank sum tests, ^∗∗∗^*p* < 0.001, ^∗∗^*p* < 0.01, and ^∗^*p* < 0.05).

In our previous work [16], we explored basic neuronal response properties in the animals enriched during early development. In the current study, we did not focus on the direct relationship between an acoustic parameter of interest and its neuronal representation but rather on stability and reproducibility of that representation, which relates to the precision of the code and possibly also to behavioral stimulus-discrimination ability. The first type of neuronal informational code tested was the rate code in which information is carried by the number of discharges during a time window. The stability of the rate code was measured in 2051 units of enriched animals (20 animals, numbers of units: 150, 150, 120, 165, 195, 165, 135, 75, 135, 120, 30, 60, 90, 45, 75, 74, 56, 84, 71, and 56) and 1714 units of control animals (17 animals, numbers of units: 107, 168, 195, 165, 165, 45, 165, 30, 45, 90, 90, 30, 120, 75, 98, 70, and 56), by computing the variances of spike counts for responses evoked by broadband noise pulses. To test the possible level-dependent behavior, the noise stimuli ranged in level from 30 to 70 dB SPL. As the neurons were previously divided to two groups according to monotonicity of their rate-intensity functions [16], the spike count variance was computed separately in these two classes of neurons. As seen in Figure 2, the enriched animals exhibit significantly lower spike count variances in the monotonic neurons, indicating a higher stability of responses at all tested intensities (Wilcoxon rank sum tests, *p* < 0.001 at 30, 60, and 70 dB SPL; *p* < 0.01 at 40 dB SPL; and *p* < 0.05 at 50 dB SPL). In the nonmonotonic units, on the other hand, the spike count variances tend to be higher in the enriched animals; however, the differences are not statistically significant (Wilcoxon rank sum tests, *p* > 0.7 at all intensities).

Signal detection theory [20, 21] states that the smallest detectable change of firing rate depends not only on the spike count variance but also on the differences of firing rates evoked by the two sounds of different intensities. For this reason, we also computed detection distances where possible by combining the current data with the firing rate data from [16]. Figure 2 illustrates that in the monotonic neurons, the detection distances are significantly higher in the enriched animals at 40 and 50 dB SPL (Wilcoxon rank sum tests, *p* < 0.001 at 40 dB SPL; and *p* < 0.01 at 50 dB SPL), while in nonmonotonic neurons, the detection distances do not differ between the animal groups (Wilcoxon rank sum tests, *p* > 0.3 at all intensities).

**Figure 3:**
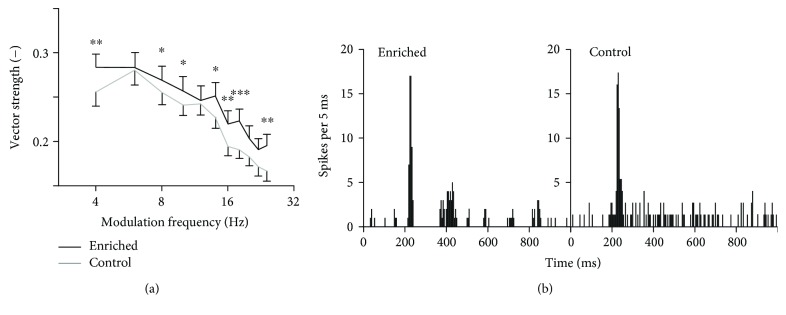
Synchronization of units with amplitude modulated noise. (a) Vector strength computed for responses to AM noise. The curves represent medians ±95% confidence intervals, and significant differences of individual data pairs are marked with asterisks (Wilcoxon rank sum tests, ^∗∗∗^*p* < 0.001, ^∗∗^*p* < 0.01, and ^∗^*p* < 0.05). (b) Typical responses of neurons from enriched and control animals to AM noise with 10 Hz modulation frequency (peristimulus time histograms).

Synchronization of the responses with temporally varying stimuli was tested first using the amplitude-modulated white noise. In this case, the fine structure of the signal was random and the neurons synchronized only with the envelope. We analyzed 593 units of enriched animals (8 animals, numbers of units: 96, 48, 80, 65, 64, 96, 80, and 64) and 640 units of controls (8 animals, numbers of units: 48, 96, 32, 128, 80, 112, 80, and 64), and only significantly phase-locking responses were considered. Typical responses to amplitude-modulated noise in the enriched and control animals are plotted in Figure 3(b). It is obvious that the neuron from the enriched animal responds with better synchronization than the control neuron. The dependence of vector strength (VS) values on the modulation frequency is plotted in Figure 3(a). The vector strength quantifies how well the individual spikes are synchronized (phase-locked) with a periodic signal. The enriched animals exhibit a significantly higher degree of synchronization at most modulation frequencies (Wilcoxon rank sum tests, significant differences occur at 4, 8, 10, 14, 16, 18, and 24 Hz). The percentages of phase-locking neurons given by the Rayleigh statistics are not significantly different in the two experimental groups (enriched: 76.4%; control: 75.3%; chi-squared test, *p* > 0.05); however, when quantifying the percentage of units with VS values higher than 0.3, the enriched animals show significantly higher values (enriched: 28%; control: 22%; chi-squared test, *p* < 0.001).

Neurons can respond to temporal modulation either by changes in their firing rate or by phase-locking to the temporal structure. To see whether the neurons with good synchronization also show changes in firing rate, we correlated the vector strength values with the firing rate in individual neurons. The results indicate that the phase-locking and changes in firing rate are largely independent, as the correlation coefficients were −0.35 in the enriched rats and −0.26 in the controls, respectively.

**Figure 4:**
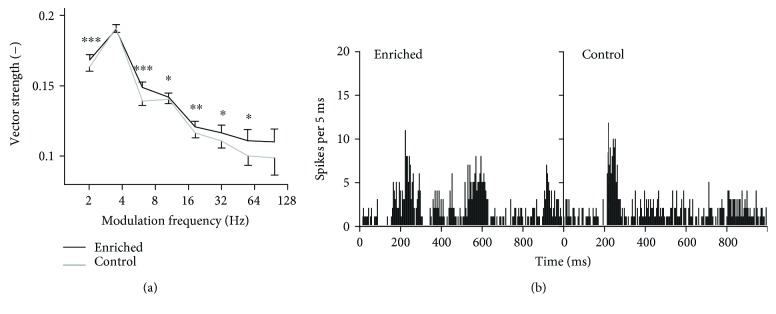
Synchronization of units with frequency modulated tones. (a) Vector strength computed for responses to FM tones. The curves represent medians ±95% confidence intervals, and significant differences of individual data pairs are marked with asterisks (Wilcoxon rank sum tests, ^∗∗∗^*p* < 0.001, ^∗∗^*p* < 0.01, and ^∗^*p* < 0.05). (b) Typical responses of neurons from enriched and control animals to FM tones with 10 Hz modulation frequency (peristimulus time histograms).

Next, synchronization with a deterministic and stationary stimulus was measured using frequency-modulated tones in 288 units of enriched animals (6 animals, numbers of units: 48, 52, 45, 47, 52, and 44) and 259 units of controls (6 animals, numbers of units: 25, 50, 38, 42, 44, and 60). Typical neuronal responses to frequency-modulated tones are depicted in Figure 4(b). Despite that the modulation period was 100 ms, the periodicity of the peristimulus time histogram indicates that the enriched neuron reacts to every second or fourth period, while the control neuron responds rather with a sustained activity. The dependence of VS values of significantly phase-locking responses on the modulation frequency is shown in Figure 4(a). As in the case of the amplitude-modulated noise, the enriched animals exhibit significantly higher VS values at most modulation frequencies (Wilcoxon rank sum tests, significant differences occur at all frequencies except 4 and 100 Hz). The percentages of phase-locking neurons are not significantly different in the two experimental groups (enriched: 42.5%; control: 42.1%; chi-squared test, *p* > 0.05); however, the percentage of units with VS values above 0.3 is higher in the enriched animals (enriched: 6%; control: 3%; chi-squared test, *p* < 0.001). As with the amplitude-modulated noise, we tried to correlate the vector strength values with the firing rate. This time the correlation is even weaker (correlation coefficients: −0.15 in the enriched rats and −0.08 in the controls, resp.); the phase-locking and changes in firing rate can be thus considered as independent for the frequency modulation.

**Figure 5:**
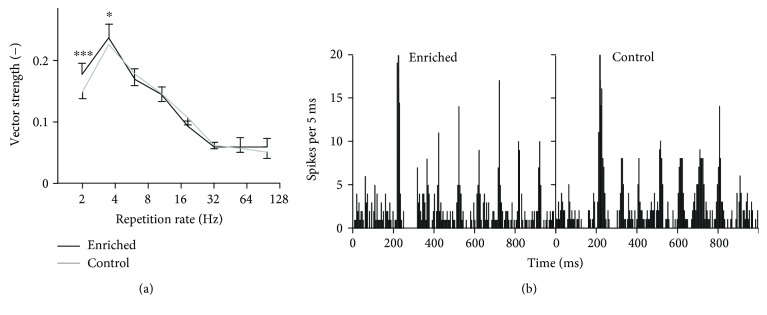
Synchronization of units with click trains. (a) Vector strength computed for responses to click trains. The curves represent medians ±95% confidence intervals, and significant differences of individual data pairs are marked with asterisks (Wilcoxon rank sum tests, ^∗∗∗^*p* < 0.001, and ^∗^*p* < 0.05). (b) Typical responses of neurons from enriched and control animals to click trains with 10 Hz repetition rate (peristimulus time histograms).

Finally, phase-locking ability to a series of clicks with different repetition rates was tested in 290 units of enriched animals (6 animals, numbers of units: 48, 56, 46, 44, 51, and 45) and 255 units of controls (6 animals, numbers of units: 25, 50, 38, 40, 42, and 60). Typical neuronal responses to click trains are depicted in Figure 5(b). Apparently, for this type of stimulus, the neurons of both groups of animals have a similar phase-locking ability: enriched animals show higher VS values only at low repetition rates, see Figure 5(a) (Wilcoxon rank sum tests, *p* < 0.001 at 2 Hz and *p* < 0.05 at 4 Hz). The percentages of phase-locking neurons are not significantly different in the two experimental groups (enriched: 66.6%; control: 67.8%; chi-squared test, *p* > 0.05); however, the percentage of units with VS values above 0.3 is higher in the enriched animals (enriched: 12%; control: 9%; chi-squared test, *p* < 0.01). The correlation of vector strength values and firing rates is again very low (correlation coefficients: 0.16 in the enriched rats and 0.19 in the controls, resp.); the phase-locking and changes in firing rate can be thus considered as independent.

**Figure 6:**
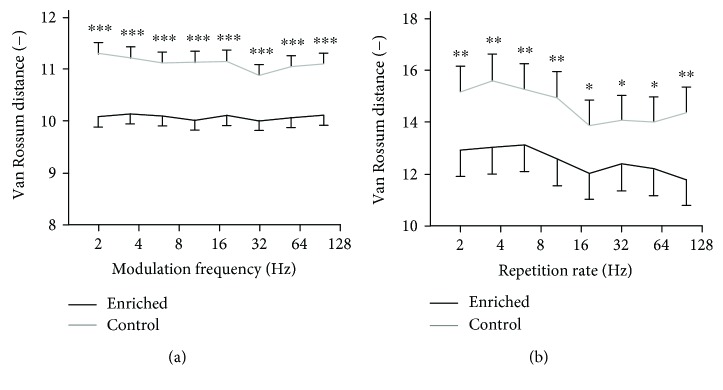
van Rossum distances of responses to a repetitive stimulus computed for responses to frequency-modulated tones (a) and click trains (b). The curves represent medians ±95% confidence intervals, and significant differences of individual data pairs are marked with asterisks (Wilcoxon rank sum tests, ^∗∗∗^*p* < 0.001, ^∗∗^*p* < 0.01, and ^∗^*p* < 0.05).

The vector strength measure evaluates the position of each spike within the period of the signal. Simply put, to achieve high VS, most spikes have to lie near a specific phase of the periodic signal. The VS thus indicates the ability of the neuron to capture the periodicity of the signal. However, it means neither that a spike has to appear in every signal period nor that the response patterns as a whole have to be similar. In order to see whether the individual neurons give response patterns that are similar to each other, we computed the van Rossum distances of different responses to the same stimulus. As the noise-based stimuli are inherently random, we used the deterministic frequency-modulated tones and click trains. For both types of stimuli, the van Rossum distances were nearly independent on the modulation frequency or repetition rate, as seen in Figure 6. Clearly, the enriched animals express a higher degree of similarity of responses across multiple stimulus repetitions both for the FM tones (Wilcoxon rank sum tests, *p* < 0.001 at all modulation frequencies) and for the series of clicks (Wilcoxon rank sum tests, *p* < 0.05 at 18, 32, and 55 Hz repetition rates, *p* < 0.01 elsewhere).

## 4. Discussion

The current study adds another piece to the mosaic of knowledge about environmental effects during hearing development. It is known from previous studies that maturation of the central auditory system is shaped by input to the developing nuclei. The sound-evoked activity controls the refinements of the structure of neuronal network [22–25], which in turn results in functional refinements and maturation of neuronal responsiveness and representation of the stimulus [4, 6, 9, 26]; hence, altering the input will also alter the development of the system. Despite considerable uncertainty as to what effect a specific stimulation will have, it appears that the resulting state of the system relates to spectral and temporal characteristics of the acoustic input. Studies that used a simple and basic stimulus often report an expanded representation of that stimulus in the AC [3, 4], while a complex environment may lead to nonspecific changes spanning the entire hearing range [6, 11, 16, 27]. The issue is mostly explored by evaluation of the neuronal receptive fields and tonotopical arrangement—a description of the system based on averaging multiple responses over time. Here, we show that besides modifications of integrated receptive fields, environmental conditions also affect the detailed structure of the spiking patterns.

Most of the works investigating developmental plasticity exposed the animals during a period starting shortly before the onset of hearing (often on P7 or P9) and lasting for two to four weeks [3, 4, 7, 12, 13, 28, 29]. The period between P11 and P13 is often stressed out as the most important period for the development of AC, and in particular, for tonotopy. However, numerous works showed that also exposure which starts markedly later (e.g., on P21) profoundly influences development of the auditory system [11, 30–34]. Our recent works have focused on the period after P14 in which the basic tonotopical arrangement is mostly final and the sensory experience shapes other properties of the auditory system: sensitivity, frequency selectivity, sound level coding, or morphology of AC neurons [6, 8, 10, 22, 35–37]. Previous studies reported that the sensitive phase of postnatal development (the so-called critical period) is not homogenous and uniform but rather that it may be divided in a series of distinct time windows during which different features of the auditory system develop consecutively [2, 38, 39]. From this viewpoint, the sound exposure used in most of the works on developmental plasticity covers several phases of the critical period. Hence, it is not currently possible to clearly identify which phase of the critical period is responsible for maturation of the spiking patterns, as reported in the current study.

In our experiments, we are interested in global changes that would not be stimulus specific but rather influence general response characteristics of auditory neurons. To accomplish this, we have developed a scenario in which the neuronal responses are mostly measured using different stimuli than those contained in the acoustic-enriching environment. This approach makes the results independent on the chosen enriching environment and makes them more comparable to other studies. In our previous studies on the inferior colliculus [6] and auditory cortex [16], we showed that developmentally enriched animals exhibited permanent changes of neuronal responsiveness; in particular, the neurons of the enriched animals were on average more sensitive (lower excitatory thresholds), more frequency selective (higher quality factor), and less nonmonotonic with respect to intensity coding (lower percentage of nonmonotonic rate-level functions). The changes were present across the entire range of tonotopical sites and were not directly linked to the properties of the acoustical stimulation. Extending and confirming these data, the current results show that an acoustical enrichment during the sensitive phase of postnatal development also influences the stability and precision of the rate code and the accuracy and reproducibility of the temporal code in the auditory cortex neurons. Importantly, the effect can still be seen in adult animals; that is, the developmental intervention introduces long-lasting consequences rather than temporary alterations which disappear shortly after the return of the organism to standard conditions.

Our experimental paradigm involves active discrimination of randomly occurring acoustic stimuli embedded in a complex modulated background noise. Previous studies show that the effects of a given acoustic stimulation may be different depending on the attention of the subjects [6, 14, 40]. Our idea was to attract the animals' attention to the acoustic environment and to motivate them to listen to it. It is conceivable that the outcomes presented in the current study are a consequence of a combination of the acoustic stimulation and the active behavioral task.

As a first outcome, we presented the improved stability of the rate-based stimulus representation. The principle of the rate code lies in representation of quantity (sound level in our case) by the number of neuronal discharges, or, when normalized by time, the spike-rate. Due to the inherent randomness and variability of neuronal firing, the evoked spike count is different for each stimulus presentation; the information on sound level is thus encoded in a mean spike count averaged over several stimulus repetitions. Stimuli of different sound levels will result in different evoked spike counts; however, for the difference to be detectable, it must be large enough not to be masked by the spiking variability [21, 41]. In the enriched animals, reduced spike count variances were observed, which suggests an improved discrimination ability of sound level changes. On the other hand, the neurons in the enriched animals may have lower evoked spike counts at the same time [16]. With regard to level discrimination ability, these two facts operate in opposite directions; thus to better quantify the level discrimination ability, detection distance (sometimes also called sensitivity index) was computed. We found that detection distances were larger in the enriched animals. According to signal detection theory [20, 21], this fact corresponds to lower theoretical just-noticeable differences of sound level, that is, better level discrimination ability. Nevertheless, to determine the influence on subjective perception of sound, an additional behavioral experiment would be necessary. Interestingly, the described changes occurred only in neurons with monotonic rate-intensity functions; detection distances of nonmonotonic neurons did not differ significantly between enriched and control animals. This indicates that monotonic and nonmonotonic neurons might play different roles with respect to sound level coding.

Information carried by the firing rate is usually based on integration (or averaging) across the interval of around 100 ms long [42, 43], thus discarding the information on events occurring at shorter time scales. Unlike the rate-based code, temporal coding employs precise positioning of individual spikes in time [44], which may potentially carry larger information content than the rate code, and furthermore, the information is conveyed almost immediately [45]. Considering also the neuronal populations, if a group of neurons projecting to another neuron produces spike patterns that are well-aligned in time, the higher synchrony will improve the detection of the signal. We studied the temporal acuity using two measures: the vector strength quantifying the synchronization of spikes with sound period and the van Rossum distance quantifying the similarity of individual-evoked patterns.

The neurons in the enriched animals exhibited an improved synchronization with temporally structured stimuli—amplitude-modulated noise, frequency-modulated tones, and click trains. The observed higher vector strength values indicate that the evoked spikes are positioned more precisely within the period of the signal modulation (in the case of amplitude-modulated noise and frequency-modulated tones) or repetition (in the case of click trains). The dependence of vector strength on the modulation frequency or repetition rate is similar in both animal groups and has the character of a shallow bandpass filter. In the case of the amplitude-modulated noise, the modulation VS function peaks around 6 Hz while for the FM tones and click trains, the peak lies slightly lower, around 4 Hz. These values are somewhat lower than those observed by Chang et al. [26] and Zhou and Merzenich [17], around 10 to 12 Hz; the difference is probably due to different measuring stimuli (noise or tone pulses) and different time of exposure. The proportion of significantly phase-locking neurons is not different in the two groups; however, the percentage of neurons with high VS values is significantly higher in the enriched rats. Taken together, the AEE used in the current study did not change the overall VS tuning properties of AC neurons; it instead increased the temporal acuity of the responses. This fact suggests that the enriched animals might have a better ability to detect and discriminate temporally varying stimuli; furthermore, the regularity of firing patterns may influence neural computations [46]. In our previous study [16], we showed that the enriched animals exhibited also different shapes or shifts of modulation-transfer functions, which again shows that both rate and temporal codes were affected by the enrichment.

Both the number of evoked potentials and the vector strength represent measures that are based on summation and averaging or pooling over time; hence, they provide limited information on the temporal spiking patterns. Yet, the reproducibility and self-consistency of firing patterns as a response to a given stimulus may play an important role in stimulus detection and discrimination tasks, particularly in complicated listening conditions such as a noisy environment. The smaller van Rossum distances of firing patterns to a repetitive stimulus in the enriched group indicate that the individual neuronal responses are more similar to each other, thus encoding more precisely or reliably the respective stimulus. This fact may relate to the experimental paradigm in which the enriched animals were motivated to discriminate several similar stimuli to obtain a reward. Smaller van Rossum distances after an acoustic training have also been observed by Cheng et al. [19].

While the developmental alterations of neuronal receptive fields after enrichment as reported, for example, in Bureš et al. [6, 16], can be explained by influencing the formation of detailed topographic maps and neuronal projections, such structural changes are probably not completely responsible for the changes of reliability and precision of spiking patterns. Partly, the changes in discharge characteristics can be influenced by an altered function of inhibitory circuits, which may develop after neonatal sound exposure [8, 10, 29, 47]. However, the observed improvements most likely result from changes at the subcellular and synaptic level. Tao et al. [48] reported that amplitude and timing of events at the synaptic input highly influence the generation and timing of output spikes in the auditory cortex. According to Rodriguez-Molina and Aertsen [49], timing and reliability of firing patterns depend on the synaptic potential kinetics, temporal jitter of excitatory inputs, and background synaptic noise. Zhou et al. [50] found that the temporal diversity of responses in the dorsal cochlear nucleus is dependent on the properties of the excitatory and inhibitory inputs and excitatory/inhibitory balance. It thus appears that the environmental conditions during the critical period of development also influence the properties of the synaptic transmission. As learning, training, and attention were shown to affect neural synchrony [19, 51] and modulate neural coding of attended stimuli [52], the stimulus discrimination task which was included in the enriching environment of the current study probably also contributed to the presented results.

## 5. Conclusions

Reliability and precision of neuronal firing is a key aspect determining the ability to detect and/or discriminate acoustic signals. Here, we have shown that this aspect is influenced by the acoustic environment that encompasses the organism during its postnatal development. An enriched environment presented shortly after the onset of hearing has the power to improve the stability of evoked cortical responses and to lower the variance of spike rates in response to a repetitive stimulus. Furthermore, the temporal acuity of individual spikes expressed by synchronization and similarity indexes (vector strength and van Rossum distance, resp.) was significantly amended compared to the control animals. The changes were retained to adulthood, suggesting that they had their origin in developmental processes, presumably shaping the properties of excitatory and inhibitory synaptic transmission. As neuronal synchrony and ability to capture the temporal changes of an acoustic stimulus are important prerequisites of speech understanding, particularly in demanding listening conditions, the results of this study again emphasize the importance of a rich and stimulating acoustic environment during hearing development.

## Abbreviations

AC: Auditory cortex
AEE: Acoustically enriched environment
AM: Amplitude-modulated
CP: Critical period
FM: Frequency-modulated
IC: Inferior colliculus
SPL: Sound pressure level
VS: Vector strength.

## References

[1] Rybalko N., Syka J. (2001). Susceptibility to noise exposure during postnatal development in rats. *Hearing Research*.

[2] Insanally M. N., Kover H., Kim H., Bao S. (2009). Feature-dependent sensitive periods in the development of complex sound representation. *Journal of Neuroscience*.

[3] de Villers-Sidani E., Chang E. F., Bao S., Merzenich M. M. (2007). Critical period window for spectral tuning defined in the primary auditory cortex (A1) in the rat. *Journal of Neuroscience*.

[4] Zhang L. I., Bao S., Merzenich M. M. (2001). Persistent and specific influences of early acoustic environments on primary auditory cortex. *Nature Neuroscience*.

[5] Zhou X., Nagarajan N., Mossop B. J., Merzenich M. M. (2008). Influences of un-modulated acoustic inputs on functional maturation and critical-period plasticity of the primary auditory cortex. *Neuroscience*.

[6] Bureš Z., Bartošová J., Lindovský J., Chumak T., Popelář J., Syka J. (2014). Acoustical enrichment during early postnatal development changes response properties of inferior colliculus neurons in rats. *European Journal of Neuroscience*.

[7] Chang E., Merzenich M. (2003). Environmental noise retards auditory cortical development. *Science*.

[8] Bureš Z., Grécová J., Popelář J., Syka J. (2010). Noise exposure during early development impairs the processing of sound intensity in adult rats. *European Journal of Neuroscience*.

[9] Bureš Z., Popelář J., Syka J. (2017). The effect of noise exposure during the developmental period on the function of the auditory system. *Hearing Research*.

[10] Grécová J., Bureš Z., Popelář J., Šuta D., Syka J. (2009). Brief exposure of juvenile rats to noise impairs the development of the response properties of inferior colliculus neurons. *European Journal of Neuroscience*.

[11] Engineer N. D., Percaccio C. R., Pandya P. K., Moucha R., Rathbun D. L., Kilgard M. P. (2004). Environmental enrichment improves response strength, threshold, selectivity, and latency of auditory cortex neurons. *Journal of Neurophysiology*.

[12] Oliver D. L., Izquierdo M. A., Malmierca M. S. (2011). Persistent effects of early augmented acoustic environment on the auditory brainstem. *Neuroscience*.

[13] Miyakawa A., Gibboni R., Bao S. (2013). Repeated exposure to a tone transiently alters spectral tuning bandwidth of neurons in the central nucleus of inferior colliculus in juvenile rats. *Neuroscience*.

[14] Polley D., Steinberg E., Merzenich M. (2006). Perceptual learning directs auditory cortical map reorganization through top-down influences. *Journal of Neuroscience*.

[15] Percaccio C. R., Pruette A. L., Mistry S. T., Chen Y. H., Kilgard M. P. (2007). Sensory experience determines enrichment-induced plasticity in rat auditory cortex. *Brain Research*.

[16] Pysanenko K., Bureš Z., Lindovský J., Syka J. (2018). The effect of complex acoustic environment during early development on the responses of auditory cortex neurons in rats. *Neuroscience*.

[17] Zhou X., Merzenich M. M. (2008). Enduring effects of early structured noise exposure on temporal modulation in the primary auditory cortex. *Proceedings of the National Academy of Sciences*.

[18] van Rossum M. C. W. (2001). A novel spike distance. *Neural Computation*.

[19] Cheng Y., Jia G., Zhang Y. (2017). Positive impacts of early auditory training on cortical processing at an older age. *Proceedings of the National Academy of Sciences of the United States of America*.

[20] Green D. M., Swets J. A. (1966). *Signal Detection Theory and Psychophysics*.

[21] Steven Colburn H., Carney L. H., Heinz M. G. (2003). Quantifying the information in auditory-nerve responses for level discrimination. *Journal of the Association for Research in Otolaryngology*.

[22] Ouda L., Burianová J., Balogová Z., Lu H. P., Syka J. (2016). Structural changes in the adult rat auditory system induced by brief postnatal noise exposure. *Brain Structure and Function*.

[23] Kandler K., Gillespie D. C. (2005). Developmental refinement of inhibitory sound-localization circuits. *Trends in Neurosciences*.

[24] Sanes D. H., Takács C. (1993). Activity-dependent refinement of inhibitory connections. *European Journal of Neuroscience*.

[25] Lu H. P., Syka J., Chiu T. W., Poon P. W. F. (2014). Prolonged sound exposure has different effects on increasing neuronal size in the auditory cortex and brainstem. *Hearing Research*.

[26] Chang E. F., Bao S., Imaizumi K., Schreiner C. E., Merzenich M. M. (2005). Development of spectral and temporal response selectivity in the auditory cortex. *Proceedings of the National Academy of Sciences*.

[27] Jiang C., Xu X., Yu L., Xu J., Zhang J. (2015). Environmental enrichment rescues the degraded auditory temporal resolution of cortical neurons induced by early noise exposure. *European Journal of Neuroscience*.

[28] de Villers-Sidani E., Simpson K. L., Lu Y.-F., Lin R. C. S., Merzenich M. M. (2008). Manipulating critical period closure across different sectors of the primary auditory cortex. *Nature Neuroscience*.

[29] Dorrn A. L., Yuan K., Barker A. J., Schreiner C. E., Froemke R. C. (2010). Developmental sensory experience balances cortical excitation and inhibition. *Nature*.

[30] Percaccio C. R., Engineer N. D., Pruette A. L. (2005). Environmental enrichment increases paired-pulse depression in rat auditory cortex. *Journal of Neurophysiology*.

[31] Bose M., Muñoz-llancao P., Roychowdhury S. (2010). Effect of the environment on the dendritic morphology of the rat auditory cortex. *Synapse*.

[32] Xu J., Yu L., Cai R., Zhang J., Sun X. (2009). Early auditory enrichment with music enhances auditory discrimination learning and alters NR2B protein expression in rat auditory cortex. *Behavioural Brain Research*.

[33] García-Estrada J., Ruvalcaba-Delgadillo Y., Luquín S. (2015). Early-life exposure to noise reduces mPFC astrocyte numbers and T-maze alternation/discrimination task performance in adult male rats. *Noise & Health*.

[34] Nichols J. A., Jakkamsetti V. P., Salgado H., Dinh L., Kilgard M. P., Atzori M. (2007). Environmental enrichment selectively increases glutamatergic responses in layer II/III of the auditory cortex of the rat. *Neuroscience*.

[35] Rybalko N., Bureš Z., Burianová J., Popelář J., Grécová J., Syka J. (2011). Noise exposure during early development influences the acoustic startle reflex in adult rats. *Physiology & Behavior*.

[36] Rybalko N., Chumak T., Bureš Z., Popelář J., Šuta D., Syka J. (2015). Development of the acoustic startle response in rats and its change after early acoustic trauma. *Behavioural Brain Research*.

[37] Šuta D., Rybalko N., Shen D.-W., Popelář J., Poon P. W. F., Syka J. (2015). Frequency discrimination in rats exposed to noise as juveniles. *Physiology & Behavior*.

[38] Insanally M. N., Albanna B. F., Bao S. (2010). Pulsed noise experience disrupts complex sound representations. *Journal of Neurophysiology*.

[39] Polley D. B., Thompson J. H., Guo W. (2013). Brief hearing loss disrupts binaural integration during two early critical periods of auditory cortex development. *Nature Communications*.

[40] Fritz J., Shamma S., Elhilali M., Klein D. (2003). Rapid task-related plasticity of spectrotemporal receptive fields in primary auditory cortex. *Nature Neuroscience*.

[41] Silver R. A. (2010). Neuronal arithmetic. *Nature Reviews Neuroscience*.

[42] Fastl H., Zwicker E. (2007). *Psychoacoustics: Facts and Models*.

[43] Bureš Z., Maršálek P. (2013). On the precision of neural computation with interaural level differences in the lateral superior olive. *Brain Research*.

[44] Cariani P. (1999). Temporal coding of periodicity pitch in the auditory system: an overview. *Neural Plasticity*.

[45] Tiesinga P., Fellous J.-M., Sejnowski T. J. (2008). Regulation of spike timing in visual cortical circuits. *Nature Reviews Neuroscience*.

[46] Bureš Z. (2012). The stochastic properties of input spike trains control neuronal arithmetic. *Biological Cybernetics*.

[47] Takesian A. E., Kotak V. C., Sanes D. H. (2012). Age-dependent effect of hearing loss on cortical inhibitory synapse function. *Journal of Neurophysiology*.

[48] Tao C., Zhang G., Zhou C. (2016). Synaptic basis for the generation of response variation in auditory cortex. *Scientific Reports*.

[49] Rodriguez-Molina V. M., Aertsen A., Heck D. H. (2007). Spike timing and reliability in cortical pyramidal neurons: effects of EPSC kinetics, input synchronization and background noise on spike timing. *PLoS One*.

[50] Zhou M., Li Y.-T., Yuan W., Tao H. W., Zhang L. I. (2015). Synaptic mechanisms for generating temporal diversity of auditory representation in the dorsal cochlear nucleus. *Journal of Neurophysiology*.

[51] Yokota R., Aihara K., Kanzaki R., Takahashi H. (2015). Learning-stage-dependent plasticity of temporal coherence in the auditory cortex of rats. *Brain Topography*.

[52] Jaaskelainen I. P., Ahveninen J. (2014). Auditory-cortex short-term plasticity induced by selective attention. *Neural Plasticity*.

